# Supplementary figures and images for: Methylation of SRD5A2 promoter predicts a better outcome for castration-resistant prostate cancer patients undergoing androgen deprivation therapy
Source: PLoS One. 2020 Mar 5;15(3):e0229754. doi: 10.1371/journal.pone.0229754 (PMC7058338; doi:10.1371/journal.pone.0229754)

## Slide 1
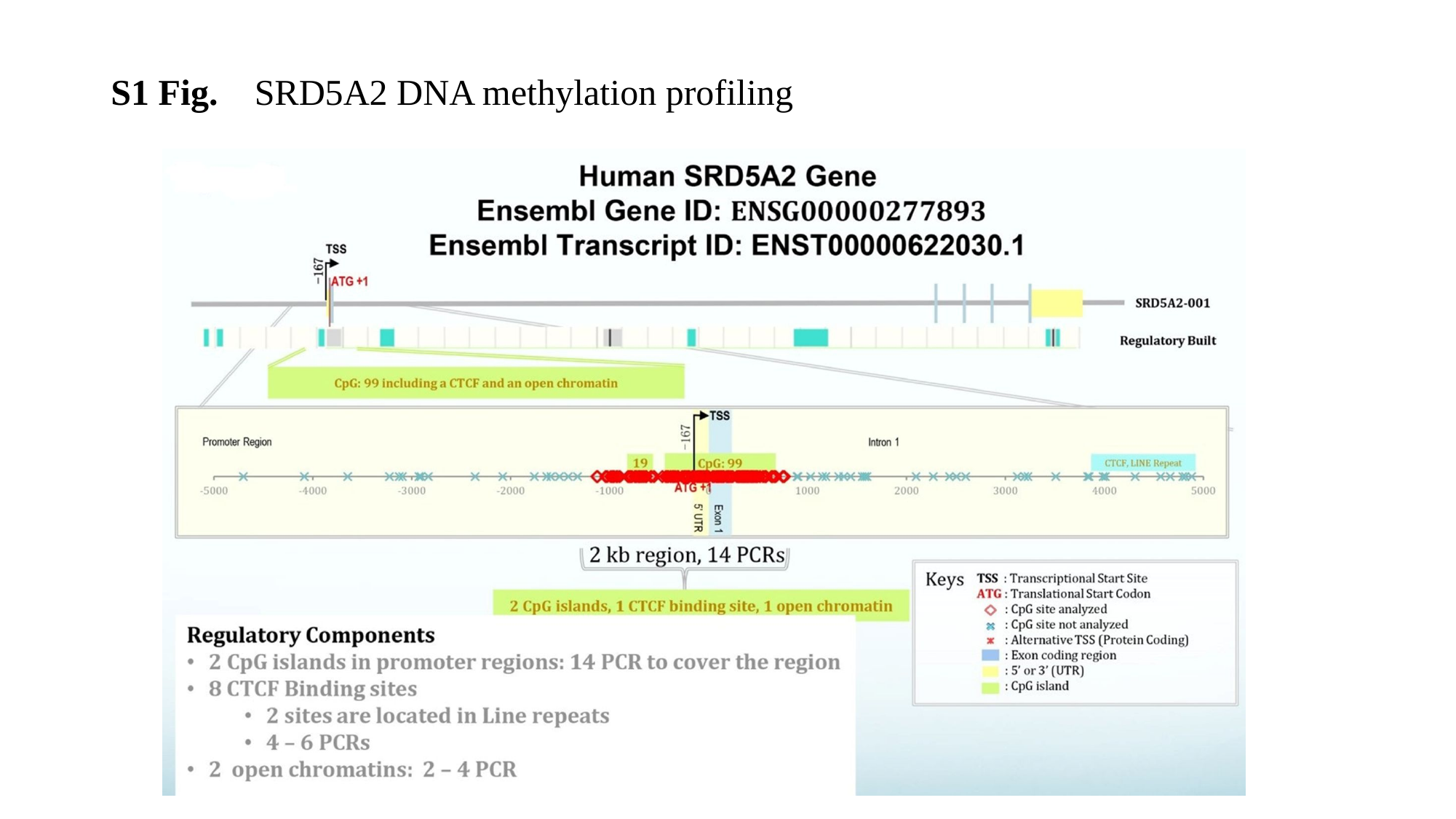

# S1 Fig. SRD5A2 DNA methylation profiling

Supplement: S1 Fig — (PPTX) [file pone.0229754.s001.pptx]

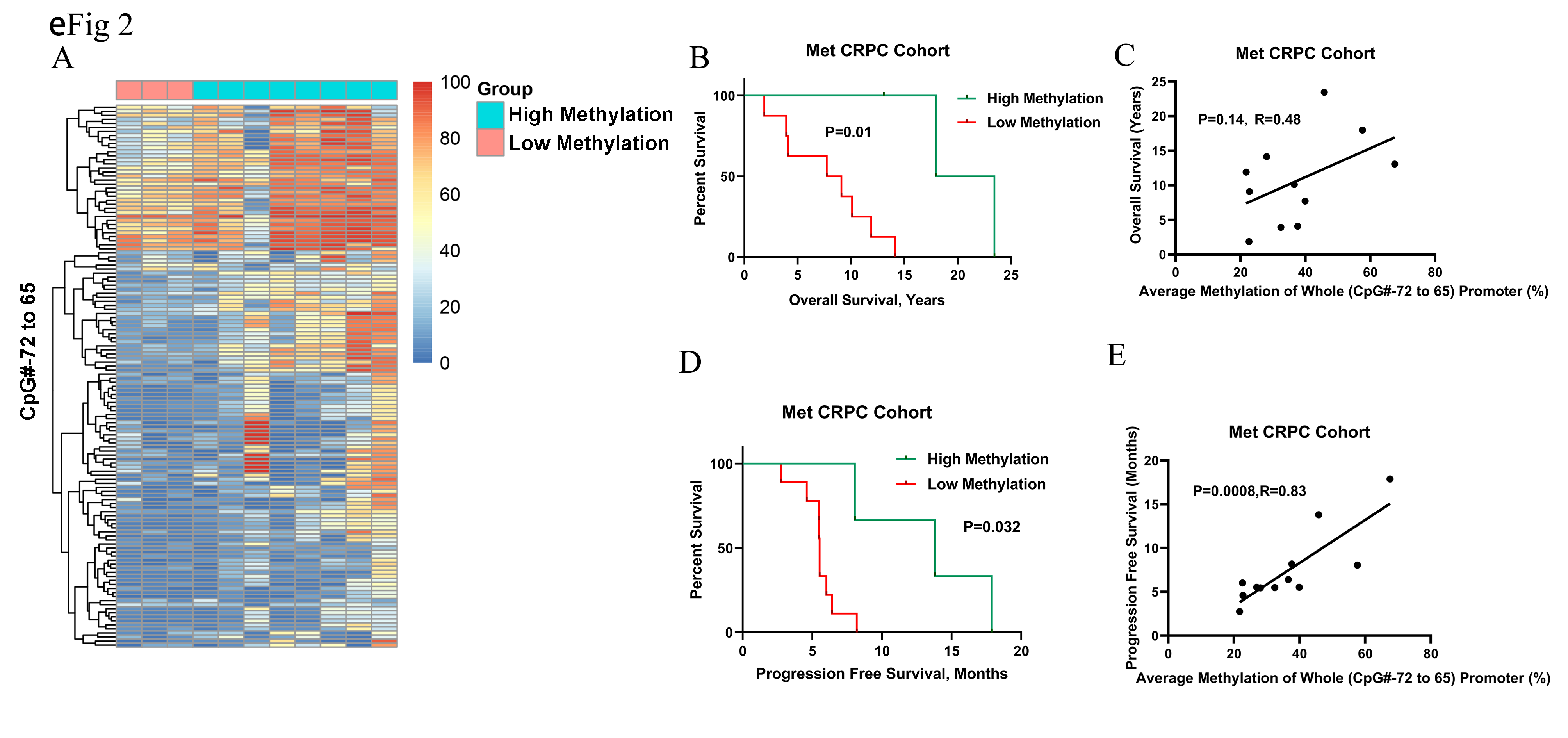

Supplement: S2 Fig — Patients were divided into a hypermethylation group and a hypomethylation group with a cutoff value of 40.0%, based on the average methylation level of all methylation sites. (A) Unsupervised cluster analysis of the ratio of SRD5A2 promoter methylation on all CpG methylation sites. (B) The difference of OS between the hypermethylation group and the hypomethylation group. (C) The correlation between methylation level and OS. (D & E) Patients were divided into a hypermethylation group and a hypomethylation group with a cutoff value of 32.5%, based on the average methylation level of all methylation sites. (D) The difference of PFS between hypermethylation group and hypomethylation group. (E) The correlation between SRD5A2 promoter methylation level and PFS. (TIF) [file pone.0229754.s002.tif]

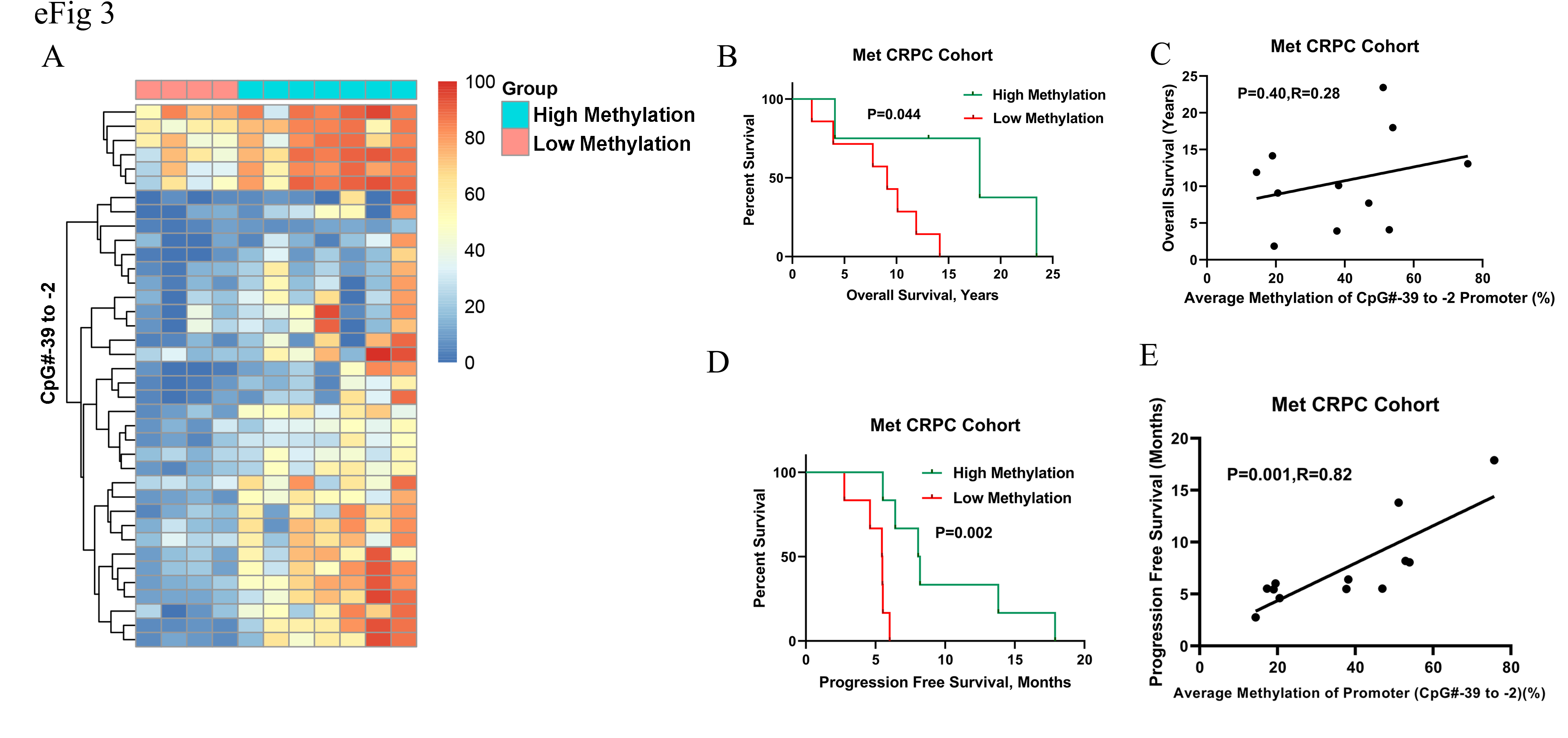

Supplement: S3 Fig — (A) Unsupervised cluster analysis of the ratio of SRD5A2 promoter methylation. (B) The difference of OS between the two groups. (C) The correlation between methylation level and OS. (D & E) Patients were divided into a hypermethylation group and a hypomethylation group with a cutoff value of 37.8%, based on the average methylation level of specific CpG sites (CpG# -39 to -2). (D) The difference of PFS between the two groups. (E) The correlation between methylation level and PFS. (TIF) [file pone.0229754.s003.tif]
