# Supplementary material for: Methylation of SRD5A2 promoter predicts a better outcome for castration-resistant prostate cancer patients undergoing androgen deprivation therapy
Source: PLoS One. 2020 Mar 5;15(3):e0229754. doi: 10.1371/journal.pone.0229754 (PMC7058338; doi:10.1371/journal.pone.0229754)
Supplement: S1 Material and methods — (DOCX) [file pone.0229754.s004.docx]

**Supporting information**

**Supporting material and methods**

**Retrospection of medical records**

Medical records of those CRPC patients were reviewed to retrospectively collect clinical and pathological data, which included patient age, date of first diagnosis with PCa, date of first diagnosis with CRPC, baseline Gleason score (GS) and PSA at the diagnosis of CRPC, time to progression, PFS, and date of death (if available). The OS was defined as time from registration to death or the last follow-up date on which patients were reported to be alive. The PFS was defined as time from registration to progression or death, whichever came first, or the last follow-up date on which patients were reported to be alive without disease progression. The OS and PFS rates were estimated using the Kaplan-Meier method. In the Local CRPC cohort, patients had a median age of 74 years (Interquartile Range, IQR, 70-82 years). The median PSA level before the TURP were 40.5 ng/ml (IQR 7.9-65.8). The GS was as follows: 2 cases of 3+4, 4 cases of 4+3, 6 cases of GS8 (3+5, 5+3, 4+4), 21 cases of GS9 (4+5 and 5+4) and 9 cases of GS10. For the Met CRPC cohort, details of the trial and participant characteristics are described elsewhere [37]. In brief, the median PSA at initiation of therapy was 28.8 ng/mL, and after 2 cycles of abiraterone, the median PSA declined to 10.9 ng/mL.

**DNA methylation test: Targeted Next-Gen Bisulfite Sequencing (tNGBS) technique**

Gene sequence information: The *SRD5A2* gene promoter has a total of 68,588 bases, and the DNA methylation profiling (Chromosome 2: 31,522,480-31,581,067) includes 5,000 bases upstream and downstream of the gene promoter region. In order to perform methylation profiling of this gene, the regulatory regions from the Ensembl Regulatory Built and the CpG islands based on the CpG islands identified by the UCSC Genome Browser were the focus of the in silico design. About 2,201 bases surrounding the transcriptional sites were used for in silico designs. These designs cover the regulatory components that include 2 CpG islands, 1 CTCF binding site and one open chromatin region in the *SRD5A2* promoter regulatory region (S1 Fig).

In silico assay design: The initial assessment resulted in a total of 14 in silico designs that cover the CpG sites.

Process of testing: Genomic DNA of the prostate biopsies was extracted using QIAamp DNA Kits (Qiagen, Valencia, CA) according to the manufacturer’s instructions. DNA samples in Genomic Lysis Buffer were first processed for direct bisulfite modification using EZ DNA Methylation Direct Kit (ZymoResearch), then gradient PCR was performed at two different magnesium concentrations (1.5 mM and 3.0 mM), followed by capillary electrophoresis (CE) of the PCR products using the QIAxcel Advanced System (Qiagen). Libraries were prepared using the KAPA Library Preparation Kit for Ion Torrent platforms (Cat# KK8310) and Ion Xpress™ Barcode Adapters (Thermo Fisher), after which the library molecules were purified, quantitated, and sequenced on the Ion S5™ sequencer using Ion 530™ sequencing chips (Thermo Fisher). Finally, FASTQ files from the Ion Torrent S5 server were aligned to the local reference database using an open-source Bismark Bisulfite Read Mapper with the Bowtie2 alignment algorithm. Methylation levels were calculated in Bismark by dividing the number of methylated reads by the total number of reads.

**Immunohistochemistry**

IHC was completed as previously described.[16, 17] Briefly, tissue sections were incubated with the SRD5A2 primary antibody (PA5-42550, Thermo Fisher Scientific, Rockford, IL) at a concentration of 1:500. The secondary antibody was used at a concentration of 1:300. Pictures were taken with an Olympus CX41RF microscope. SRD5A2 expression was evaluated with the immunoreactive score (IRS) according to the percentage of positive cells (0 [negative], 1 [<10%], 2 [10-50%], 3 [51-80%] and 4 [>80%]) and staining intensity [0, 1-negative, 2-moderate, and 3-intense]).
