# Supplementary material for: Methylation of SRD5A2 promoter predicts a better outcome for castration-resistant prostate cancer patients undergoing androgen deprivation therapy
Source: PLoS One. 2020 Mar 5;15(3):e0229754. doi: 10.1371/journal.pone.0229754 (PMC7058338; doi:10.1371/journal.pone.0229754)
Supplement: S1 Protocol — (DOCX) [file pone.0229754.s005.docx]

**Protocol for Interventional Study**

**Patients**

This is a phase II, single-arm, open-label study of abiraterone and dutasteride in patients with metastatic CRPC (NCT01393730). In total, 40 patients were enrolled on the study between September 2011 and October 2012. Patients were enrolled at 3 institutions: Dana-Farber Cancer Institute (n= 27, Boston, MA), Beth Israel Deaconess Medical Center (n= 5, Boston, MA), and University of Washington (n= 8, Seattle, WA). Eligible patients had CRPC defined as disease progression despite a serum total testosterone <50 ng/dL and (i) PSA progression as defined by the Prostate Cancer Clinical Trials Working Group (PCWG) 2, (ii) soft-tissue disease progression as defined by Response Evaluation Criteria In Solid Tumors (RECIST) version 1.1, or (iii) bone disease progression as defined by PCWG2. In addition, patients had evidence of

metastases with at least one metastatic site amendable to biopsy. Patients may have had any number of prior hormonal therapies (including antiandrogens, steroids, estrogens, finasteride,

dutasteride, and ketoconazole), up to 2 previous cytotoxic therapies, radiotherapy, radiopharmaceuticals, or immunotherapy provided these were discontinued _4 weeks prior to

study treatment initiation. Other eligibility criteria included: Eastern Cooperative Oncology Group (ECOG) performance status ≤2, predefined hematologic and laboratory criteria including serum potassium ≥3.5 mmol/L, aspartate aminotransferase (AST) and alanine aminotransferase (ALT) <1.5 X institutional upper limit of normal (ULN), total serum bilirubin ≤1.5 X institutional ULN (except for participants with documented Gilbert's disease), and left ventricular ejection

fraction ≥50%. Patients were excluded if they had received prior abiraterone, had known brain metastases, uncontrolled intercurrent illness, uncontrolled hypertension (≥160 mmHg/

≥95 mmHg), liver disease, pituitary or adrenal dysfunction, clinically significant cardiovascular events or thromboembolism within 6 months, surgery or local prostatic intervention within 1 month of study treatment initiation, gastrointestinal disorders which could interfere with drug absorption, or requirement for chronic steroids greater than the equivalent of prednisone 5 mg daily. All patients provided written informed consent.

**Treatment**

Following enrollment, patients had a baseline research biopsy of a soft tissue or bone metastasis. Biopsies were performed after informed consent in the interventional radiology department. Subsequently, patients were treated with abiraterone (1,000 mg daily) and prednisone (5 mg daily) for two 4-week cycles. After this time, high-dose dutasteride (3.5 mg daily) was added. Patients continued on the 3-drug regimen until study withdrawal or radiographic disease progression. A repeat metastasis research biopsy, while patients were still on therapy, was obtained at progression in patients completing at least 4 treatment cycles.

When possible, the second biopsy was at the same site as the baseline biopsy. Although baseline and progression biopsies were mandatory, not all patients underwent biopsy at progression given lack of feasible biopsy site, clinical disease progression, or patient withdrawal. Imaging assessments occurred every 12 weeks. PSA was measured every 4 weeks, and pre- and postdutasteride PSA levels were analyzed.
