# Supplementary material for: Methylation of SRD5A2 promoter predicts a better outcome for castration-resistant prostate cancer patients undergoing androgen deprivation therapy
Source: PLoS One. 2020 Mar 5;15(3):e0229754. doi: 10.1371/journal.pone.0229754 (PMC7058338; doi:10.1371/journal.pone.0229754)
Supplement: S1 Table — (DOCX) [file pone.0229754.s006.docx]

**eTable 1-1**

| **Promoter region** | **Local CRPC cohort** | | | | | | | | |
| --- | --- | --- | --- | --- | --- | --- | --- | --- | --- |
|  | **Overall Survival** | | |  |  |  | **Progression Free Survival** | | |
| **CpG#** | **Cutoff** | **Average (Methylation)** | **Average (Years)** | **P Value** |  | **Cutoff** | **Average (Methylation)** | **Average (Months)** | **P Value** |
| -72~65 | 46.5 | 38±6.3/57±7.7 | 8±4.8/12±6.3 | **0.013** |  | 42.0 | 34.9±4.9/53.8±8.6 | 5.3±4.2/8.4±5.6 | **0.045** |
| -39~-2 | 37.9 | 26.8±8.2/55.0±10.7 | 6.4±4.4/11.3±5.8 | **0.0014** |  | 37.9 | 26.8±8.2/55±10.7 | 4.5±3.9/8.4±5.4 | **0.0032** |
| -39~65 | 27.1 | 18.9±5.8/42.6±12.4 | 7.3±4.311.1±6.1 | **0.019** |  | 27.1 | 18.9±5.8/42.6±12.4 | 5.3±3.9/8.1±5.6 | **0.038** |
