# Supplementary material for: Methylation of SRD5A2 promoter predicts a better outcome for castration-resistant prostate cancer patients undergoing androgen deprivation therapy
Source: PLoS One. 2020 Mar 5;15(3):e0229754. doi: 10.1371/journal.pone.0229754 (PMC7058338; doi:10.1371/journal.pone.0229754)
Supplement: S2 Table — (DOCX) [file pone.0229754.s007.docx]

|  | **Overall survival** | | | | | **Progression free survival** | | | | |  |  |  |  |  |  |
| --- | --- | --- | --- | --- | --- | --- | --- | --- | --- | --- | --- | --- | --- | --- | --- | --- |
|  | **Coef.** | **Std. Err.** | **t** | **P>\|t\|** | **[95% Conf. Interval]** | **Coef.** | **Std. Err.** | **t** | **P>\|t\|** | **[95% Conf. Interval]** |  |  |  |  |  |  |
| Gleason score | -0.31 | 0.441 | -0.70 | 0.487 | -1.202 to 0.583 | -0.306 | 0.425 | -0.72 | 0.476 | -1.166 to 0.554 |  |  |  |  |  |  |
| PSA | 0.002 | 0.002 | 0.29 | 0.205 | -0.001 to 0.006 | 0.0002 | 0.002 | 0.11 | 0.914 | -0.003 to 0.004 |  |  |  |  |  |  |
| SRD5A2 whole promoter (CpG#: -72 to 65) methylation | 0.164 | 0.075 | 2.18 | **0.035** | 0.012 to 0.317 | 0.098 | 0.073 | 1.35 | 0.183 | -0.049 to 0.246 |  |  |  |  |  |  |

**eTable 2.** Multivariable regression analyses

|  | **Overall survival** | | | | | **Progression free survival** | | | | |  |  |  |  |  |  |
| --- | --- | --- | --- | --- | --- | --- | --- | --- | --- | --- | --- | --- | --- | --- | --- | --- |
|  | **Coef.** | **Std. Err.** | **t** | **P>\|t\|** | **[95% Conf. Interval]** | **Coef.** | **Std. Err.** | **t** | **P>\|t\|** | **[95% Conf. Interval]** |  |  |  |  |  |  |
| Gleason score | -0.286 | 0.432 | -0.66 | 0.511 | -1.16 to 0.588 | -0.297 | 0.418 | -0.71 | 0.481 | -1.144 to 0.549 |  |  |  |  |  |  |
| PSA | 0.003 | 0.002 | 1.47 | 0.149 | -0.001 to 0.006 | 0.0004 | 0.002 | 0.21 | 0.837 | -0.003 to 0.004 |  |  |  |  |  |  |
| SRD5A2 specific promoter region (CpG#: -39 to -2) methylation | 0.127 | 0.053 | 2.42 | **0.020** | 0.021 to 0.234 | 0.08 | 0.051 | 1.57 | 0.124 | -0.023 to 0.183 |  |  |  |  |  |  |
