# Supplementary material for: Methylation of SRD5A2 promoter predicts a better outcome for castration-resistant prostate cancer patients undergoing androgen deprivation therapy
Source: PLoS One. 2020 Mar 5;15(3):e0229754. doi: 10.1371/journal.pone.0229754 (PMC7058338; doi:10.1371/journal.pone.0229754)
Supplement: S3 Table — (DOCX) [file pone.0229754.s008.docx]

| **Promoter region** | **Met CRPC Cohort** | | | | | | | | |
| --- | --- | --- | --- | --- | --- | --- | --- | --- | --- |
|  | **Overall Survival** | | |  |  |  | **Progression Free Survival** | | |
| **CpG#** | **Cutoff** | **Average (Methylation)** | **Average (Years)** | **P Value** |  | **Cutoff** | **Average (Methylation)** | **Average (Months)** | **P Value** |
| -72~65 | 40.0 | 30.0±7.0/57±10.9 | 7.86±4.28/18.18±5.19 | **0.010** |  | 40.0 | 30.0±7.0/57±10.9/57.0±10.9 | 5.6±1.4/13.3±4.9 | **0.032** |
| -39~-2 | 47.0 | 28.1±12.6/58.4±11.6 | 8.4±4.32/14.66±8.22 | **0.044** |  | 37.8 | 21.4±8.3/53.1±12.5 | 5±1.2/10±4.8 | **0.002** |
| -39~65 | 28.1 | 14.8±5.6/43.4±15.0 | 6.9±4.8/14.5±6.3 | 0.056 |  | 28.1 | 16.5±7.0/47.3±14.2 | 5.4±1.5/11.5±5.4 | **0.020** |

**eTable 1-2**
